# Phonological recoding under articulatory suppression

**DOI:** 10.3758/s13421-017-0754-8

**Published:** 2017-09-11

**Authors:** Dennis Norris, Sally Butterfield, Jane Hall, Michael P. A. Page

**Affiliations:** 1 MRC Cognition and Brain Sciences Unit, University of Cambridge, Cambridge, UK; 2 MRC Cognition and Brain Sciences Unit, Cambridge, CB2 7EF UK; 3 Department of Psychology, University of Hertfordshire, Hatfield, UK

**Keywords:** Working memory, Articulatory suppression, Phonological recoding, Memory

## Abstract

We report data from an experiment in which participants performed immediate serial recall of visually presented words with or without articulatory suppression, while also performing homophone or rhyme detection. The separation between homophonous or rhyming pairs in the list was varied. According to the working memory model (Baddeley, 1986; Baddeley & Hitch, 1974), suppression should prevent articulatory recoding. Nevertheless, rhyme and homophone detection was well above chance. However, with suppression, participants showed a greater tendency to false-alarm to orthographically related foils (e.g., GIVE–FIVE). This pattern is similar to that observed in short-term memory patients.

What is stored in verbal short-term memory (STM), and how does it get there? The standard view is that verbal STM is primarily supported by representations that are phonological in nature and, if the input is written rather than spoken, orthographic information has to be recoded into a phonological representation by engaging an articulatory control process. This, at least, is the view embodied in the working memory framework of Baddeley and Hitch (Baddeley, 1986; Baddeley & Hitch, 1974). Even though there are some diverging views (Jones, Macken, & Nicholls, 2004), most models of STM make similar assumptions (e.g., Cowan & AuBuchon, 2008; Cowan & Chen, 2008). In the working memory model, verbal STM is supported by a phonological store and an articulatory loop subsystem. The articulatory loop serves two functions: It performs subvocal rehearsal, which can offset the effects of memory decay, and it also serves to recode written input into a phonological form that can be retained in the phonological store.

This account of STM has been arrived at on the basis of two main lines of evidence. First, confusions in STM are driven mainly by phonological similarity of the items to be remembered rather than their semantic similarity (Baddeley, 1966a). For example, the list of rhyming letters B, C, E, V, and P will be harder to remember than phonologically distinct letters such as B, M, R, K, and L. Semantically based confusions generally emerge only at longer retention intervals. Second, when stimuli are presented visually, phonological confusions are reduced or eliminated by the requirement to perform concurrent articulation during input, such as repeating the word “the” aloud (Baddeley, Thomson, & Buchanan, 1975; Besner & Davelaar, 1982; Murray, 1968; Peterson & Johnson, 1971; Wilding & Mohindra, 1980). In contrast, suppression does not eliminate the effect of phonological similarity when stimuli are presented auditorily (Baddeley, Lewis, & Vallar, 1984; Levy, 1971; Murray, 1968; Peterson & Johnson, 1971). Baddley, Thomson, and Buchanan argued that whereas auditorily presented stimuli have a direct and privileged access to the phonological store, concurrent articulation (articulatory suppression) occupies the process involved in recoding the written input into a phonological form.

However, the assumption that articulatory suppression prevents visual material being phonologically recoded seems inconsistent with the widely replicated result that suppression has little effect on participants’ ability to make phonological judgments on written materials. A number of studies has shown that, while performing articulatory suppression, participants can easily and accurately judge whether pairs of words are rhymes or homophones (Besner, 1987; Besner, Davies, & Daniels, 1981; Brown, 1987; Howard & Nickels, 2005; Johnston & McDermott, 1986; Nickels, Howard, & Best, 1997; Richardson, 1987; Wilding & White, 1985). Although suppression tends to have a larger effect of rhyme than on homophone judgments, the increase in error rates on rhyme judgments is less than 10%, and this increase is often not statistically significant. This applies even when the stimuli are nonsense words, for which there is no possibility that the phonological representations can have been derived from the lexical representations rather than by recoding the orthographic input. Both Baddeley and Lewis (1981) and Brown (1987) reported a suppression effect of 5% for nonword stimuli, and this effect was significant only in Brown’s data.

The paradox, then, is that in serial recall, suppression appears to prevent phonological recoding, whereas suppression has very little effect at all when performing phonological judgments. To explain their own data showing minimal effects of suppression on rhyme and homophone judgments, Baddeley and Lewis (1981) suggested that three types of short-term buffer might be involved in reading: articulatory coding, which they referred to as the “inner voice”; acoustic coding (the “inner ear”); and visual coding (“inner eye”). However, this does not seem to address the specific question of how a phonological or “acoustic” code can be generated from written input. A simpler possibility that does not require further proliferation of short-term stores is that suppression may not block phonological recoding completely, but simply may make recoding so difficult that participants abandon it unless explicitly forced to do so by the task. This is a critical difference between serial recall and rhyme or homophone judgment tasks: Rhyme or homophone judgment tasks can only be performed by engaging in some form of phonological recoding. In serial recall, however, phonological recoding is an optional extra, and the requirement to perform simultaneous articulatory suppression may be all that is required to discourage participants from performing such recoding. Baddeley and Larsen (2003) suggested that participants may abandon the loop when recall becomes difficult—for example, when list length increases. Even in the absence of articulatory suppression, the phonological similarity effect seems to diminish as recall becomes harder because of increased list length (Baddeley, 1966a, 1966b; Baddeley & Larsen, 2003; Hall, Wilson, Humphreys, Tinzmann, & Bowyer, 1983; Hanley & Bakopoulou, 2003; Hanley & Broadbent, 1987; Johnston, 1982; Neath, Bireta, & Surprenant, 2003). One should also bear in mind that not all participants may naturally adopt a strategy of phonological recoding or rehearsal (Logie, Della Sala, Laiacona, Chalmers, & Wynn, 1996). Furthermore, Campoy (2008) and Campoy and Baddeley (2008) found that the phonological similarity effect was eliminated when participants were instructed to use a semantic encoding strategy. To the extent that some participants might need to make a positive effort to use a phonological strategy, it would seem likely that they might require little discouragement to abandon it. Here we investigated this possibility by combining the task demands of both serial recall and homophone/rhyme judgments and having participants perform homophone/rhyme judgments on words that they also had to recall. In the context of a serial recall task, would participants still be able to perform rhyme and homophone judgments with a high level of accuracy?

Additionally, the experiment manipulated the separation of rhyming or homophonous items in the input list, so as to allow us to track the time course of the availability of phonological information. The rhyming or homophonous pairs could be adjacent to each other in the list or separated by one or two unrelated intervening items. If rhyming or homophonous word pairs were separated by one or two intervening items, they would need to be held in the phonological store in order to be compared. If suppression really does impair the encoding or retention of phonological information, it should then be much harder to perform the judgment task under suppression when there are intervening items. For rhyme detection, the design was therefore 2 (Suppression) × 2 (Orthography) × 3 (Distance), and for homophone detection it was 2 (Suppression) × 3 (Distance).

## Method

### Participants

Thirty-six members of the MRC Cognition and Brain Sciences volunteer panel, 16–28 years of age, were paid a small honorarium for their participation.

### Materials

Both the rhyme and homophone judgment conditions included 90 experimental trials and ten practice trials. The lists were six items long, and all stimuli were monosyllabic. The rhyme judgment task was based on 30 pairs of rhyming words (half with matching orthography of the rime vowel and coda—e.g., *scent–rent*—and half with nonmatching orthography—e.g., *yacht–clot*) and 30 pairs of nonrhyming foil words with orthographies that matched the rime vowel and coda—for example, *give–five.* These nonrhyming orthographic foils served as a measure of how successful participants were at using a strictly phonological strategy. If they failed to use phonology and instead made their judgments on the basis of orthography, they would be expected to make false alarms to these foil trials. It is important to note that the participants were simply required to detect occasional rhymes and not to judge whether each word was a rhyme. There were also 30 filler trials in which none of the words either rhymed or shared orthography of their rime portions. The critical word pairs appeared equally often with zero, one, or two intervening words. We created three different experimental lists counterbalanced such that each critical pair appeared once at each separation.

The homophone conditions necessarily differed somewhat from the rhyme conditions, since no homophone trials could share orthography—these would be the same word. The critical stimuli consisted of 30 homophone pairs and 30 pairs of visually similar foil words that differed by one phoneme—for instance, *lick*–*like*, *dare*–*dear*, *ferry*–*fury*. There were also 30 filler trials with minimal orthographic or phonological overlap between the words in the lists. For these trials we recorded whether participants made any response at all during the list. This served as a baseline measure of the false-alarm rate. The critical pairs were largely derived from those used by Brown (1987). The critical items never occurred in initial or final position and were balanced for the number of intervening words. As in the rhyme judgment task, three experimental lists were presented, with the separation between pairs counterbalanced over lists. The experimental pairs for both rhymes and homophones are listed in the Appendix.

Note that given that the constraints on stimulus construction largely exhausted the set of possible items, no attempt was made to balance words for factors such as word frequency. Furthermore, the regularity of spelling-to-sound correspondences in the conditions necessarily varied. For example, rhyming foils (e.g., *leaf–deaf*) must all have phonologically inconsistent pronunciations. The mean SUBTLEX-UK (van Heuven, Mandera, Keuleers, & Brysbaert, 2014) word frequencies for each condition are given in the Appendix, which also gives the mean naming latencies for each condition from the English Lexicon Project (Balota et al., 2007). This can be taken as a proxy for the difficulty of deriving the phonological form of each word from its orthography.

### Procedure

Participants were tested individually in a quiet room. Written instructions explained that five words would appear on the display, one after another. In the homophone condition participants were instructed to press the response button if they saw a word that “sounds identical to another word in that sequence.” In the rhyme condition they were instructed to respond to a word that “rhymes with another word in the sequence.” After the final word, a prompt to “recall” appeared, and the participants were instructed to write the words recalled in serial order on a form provided. Also, they were told to cover their response before pressing the spacebar to initiate the next trial. Prior to all lists, there was a practice block of ten trials.

Participants were randomly assigned to each presentation list. Half the participants on each list performed an articulatory suppression task: They were asked to say the word “racket” aloud continuously, starting when they initiated each trial until they saw the screen prompt to recall. The experimenter sat in the room to monitor whether participants complied with the instructions.

The stimulus presentation, timing, and data collection were controlled by DMDX experimental software (Forster & Forster, 2003). Visual stimuli were presented on a Dell Inspiron 7000 laptop computer in uppercase Courier New 10-point font on a black background. The words appeared every 750 ms and were displayed for 650 ms, followed by a blank screen for 100 ms. Lists were presented in an independently randomized order for each participant.

## Results

**Fig. 1:**
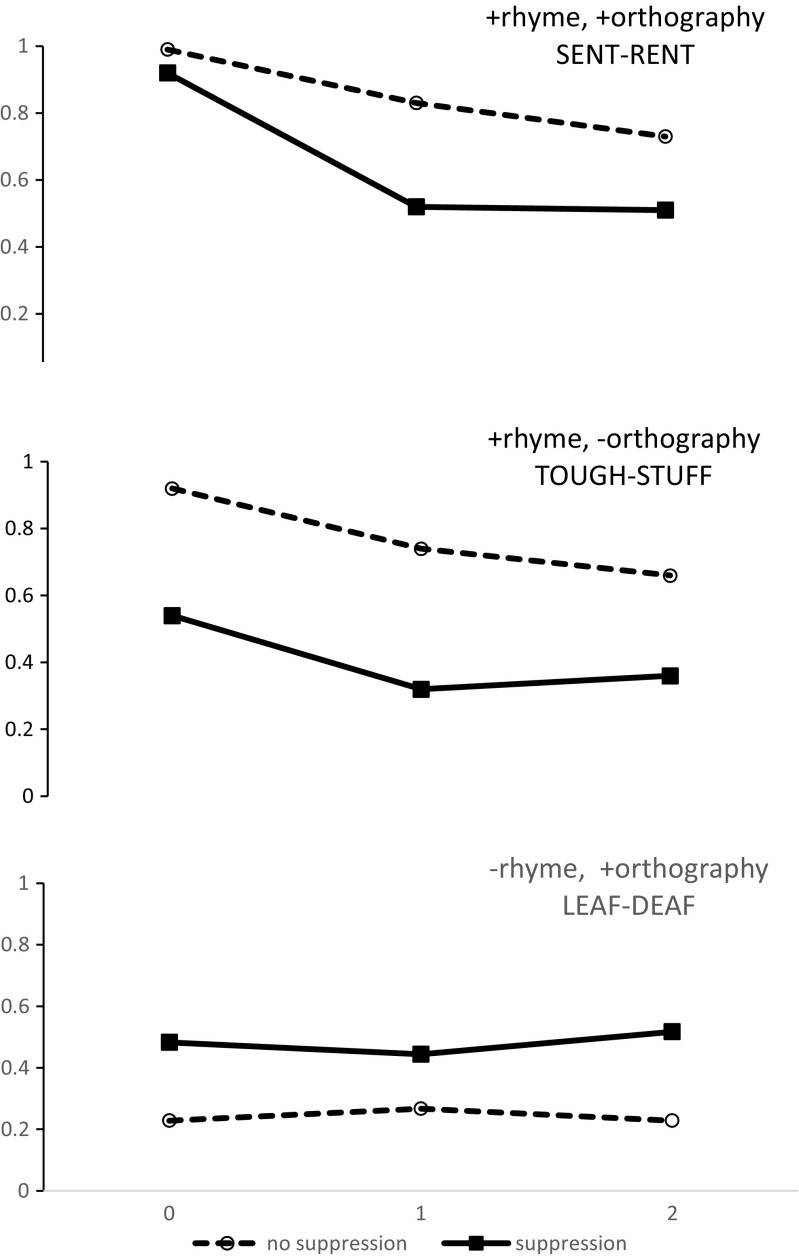
Proportions of correct detections for rhyme targets (top two panels) and false alarms to foil items (bottom panel).

**Fig. 2:**
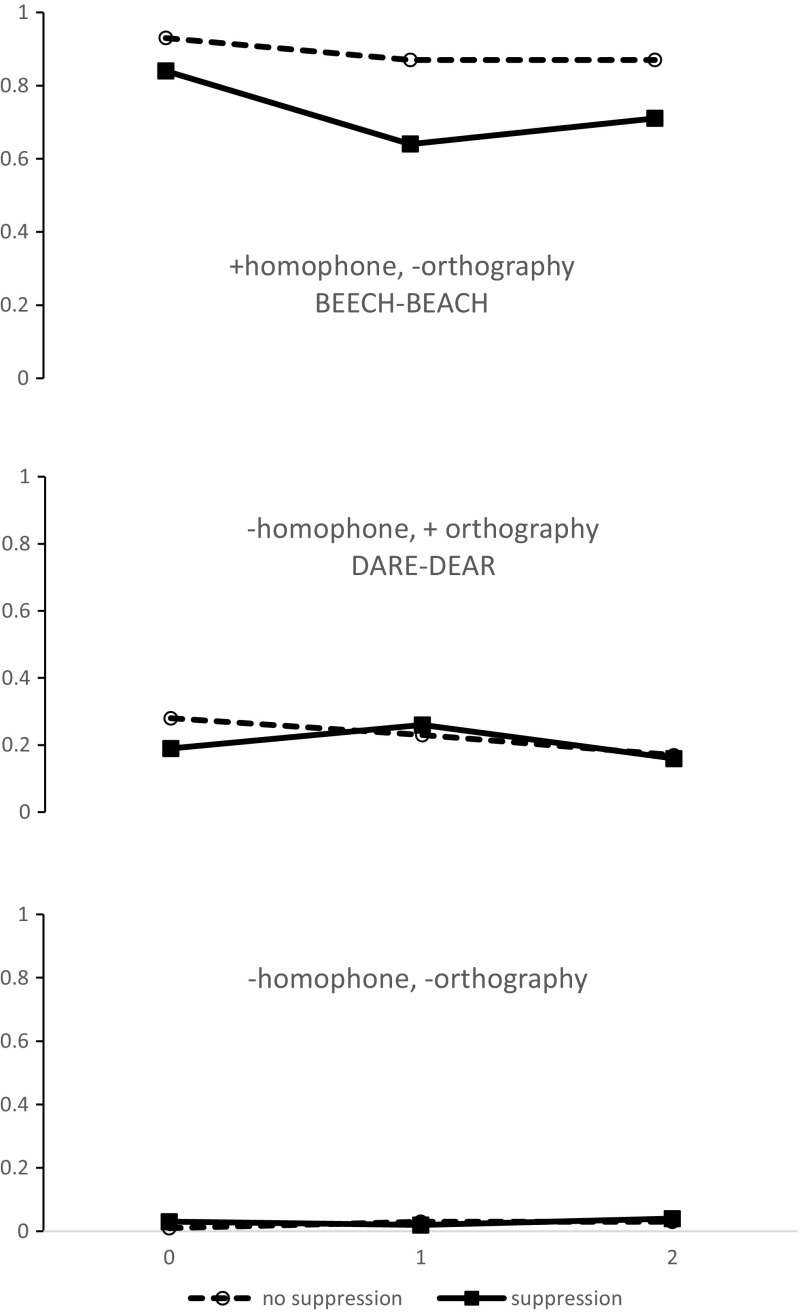
Proportions of correct detections for homophone targets (top), false alarms to foil items (middle), and responses on filler trials (bottom).

The results are shown in Figs. 1 and 2 and Table 1. Several things can be readily appreciated from the figures. First, suppression impairs the detection of both rhymes and homophones in all conditions. Second, the impairment is generally greater for rhymes than for homophones. Third, the impairment is particularly marked for rhyming items that do not share orthography; here the increase in errors can be greater than 40%. Finally, suppression increases the false-alarm rate to nonrhyming items that share orthography (*leaf–deaf*), despite the absence of any such effect on homophone detection.

**Table 1:** Numbers of words correctly recalled in each position for trials with different classes of target

Condition	Suppression	Distance		
		0	1	2
+Rhyme	No suppression	3.4	3.0	3.4
+Orthography	Suppression	1.9	1.8	1.8
SENT–RENT				
+Rhyme	No suppression	2.7	3.2	2.8
–Orthography	Suppression	1.5	1.3	1.4
TOUGH–STUFF				
–Rhyme	No suppression	3.1	3.1	3.0
+Orthography	Suppression	1.8	1.5	1.6
LEAF–DEAF				
–Rhyme	No suppression	2.9	3.0	3.1
–Orthography	Suppression	1.5	1.6	1.5
+Homophone	No suppression	3.2	3.1	3.0
–Orthography	Suppression	2.4	2.4	2.2
BEECH–BEACH				
–Homophone	No suppression	3.1	3.2	3.1
+Orthography	Suppression	2.3	2.2	2.0
DARE–DEAR				
–Homophone	No suppression	2.9	3.1	3.2
–Orthography	Suppression	2.2	2.1	2.1

The critical analyses all focus on correct detections of rhyming or homophonous trials and how these vary as a function of distance. In the case of rhyme detection, rhyming foils can either share orthography or not. We also present combined analyses of rhyme and homophone detection. These are necessarily restricted to stimuli that do not share orthography.

### Detection accuracy rhymes (Suppression × Orthography × Distance)

For rhyming targets, the main effects of suppression [*F*(1, 34) = 43.0, *MSE* = 4.33, *p* < .0001, ${\eta}_p^2$ = .56], orthography [*F*(1, 34) = 29.0, *MSE* = 1.40, *p* < .0001, ${\eta}_p^2$ = .46], and distance [*F*(2, 68) = 48.8, *MSE* = 1.65, *p* < .0001, ${\eta}_p^2$ = .59] were all significant. We also observed a significant interaction between suppression and orthography [*F*(1, 34) = 7.6, *MSE* = 0.375, *p* < .01, ${\eta}_p^2$ = .19], with suppression having a larger effect on orthographically similar than on dissimilar targets. There was also a marginally significant interaction between suppression and distance [*F*(2, 68) = 2.97, *MSE* = 0.10, *p* = .058, ${\eta}_p^2$ = .08], with the effect of suppression being larger when the rhyming items were separated.

### Homophones (Suppression × Distance)

For homophone targets we found main effects of suppression [*F*(1, 34) = 9.79, *MSE* = 0.669, *p* < .005, ${\eta}_p^2$ = .22] and distance [*F*(2, 68) = 15.1783, *MSE* = 0.179, *p* < .0001, ${\eta}_p^2$ = .31], with detection being less accurate under suppression and with increasing distance. A significant interaction between suppression and distance also emerged [*F*(2, 68) = 3.68, *MSE* = 0.0434, *p* < .05, ${\eta}_p^2$ = .1], with the suppression effect being larger with increasing distance.

### Rhymes and homophones combined (Rhyme/homophone × Suppression × Distance)

Given that all homophone pairs must necessarily have different orthographies, the combined analysis compared all of the homophone trials with the orthographically dissimilar rhyme trials.

Rhymes were detected less accurately than homophones [*F*(1, 68) = 32.48, *MSE* = 2.6, *p* < .0001, *η*_p_^2^ = .32], and there were significant main effects of suppression [*F*(1, 68) = 46.31, *MSE* = 3.71, *p* < .0001, *η*_p_^2^ = .41] and distance [*F*(2, 136) = 28.64, *MSE* = 0.66, *p* < .0001, *η*_p_^2^ = .3]. We observed a significant interaction between rhyme/homophone and suppression [*F*(1, 68) = 7.38, *MSE* = 0.59, *p* < .01, *η*_p_^2^ = .1], with suppression having a greater effect on rhyme than on homophone detection. There was also a significant interaction between rhyme/homophone and distance [*F*(2, 136) = 3.18, *MSE* = 0.073, *p* < .05, *η*_p_^2^ = .04], with distance having a greater effect on rhyme than on homophone detection. The three-way interaction between rhyme/homophone, suppression, and distance was not significant [*F*(2, 136) = 1.05].

### False alarms to rhyme foils (Suppression × Distance)

The proportion of false alarms to rhyme foils was larger under suppression [*F*(1, 34) = 15.78, *MSE* = 0.1, *p* < .0005, ${\eta}_p^2$ = .32]. We found no effect of distance (*F* < 1) and no interaction between distance and suppression [*F*(2, 68) = 1.34, *p* > .1].

In a separate analysis, we compared orthographically related rhymes with orthographically related rhyme foils under suppression. These two conditions both have orthographic overlap, but in only one do the words rhyme. This would tell us whether participants were still making use of phonology under suppression. There were more correct responses to rhymes than error responses to foils [*F*(1, 17) = 12.65, *MSE* = 0.06, *p* < .005, ${\eta}_p^2$ = .43], and this difference decreased with increasing distance [*F*(1, 34) = 13.05, *MSE* = 0.04, *p* < .0001, ${\eta}_p^2$ = .43]. We also found a main effect of distance, attributable to the decrease in correct detections of rhymes with distance [*F*(1, 34) = 25.83, *MSE* = 0.02, *p* < .0002, ${\eta}_p^2$ = .60].

### Homophone foils (Suppression)

There were hardly any false alarms to lists with no orthographically similar foils (mean = 2.7%). That is, participants’ baseline level of random responding was near zero. The proportion of false alarms to orthographically related foils decreased with greater separation [*F*(2, 62) = 5.16, *MSE* = 0.075, *p* < .01, ${\eta}_p^2$ = .13] but was not influenced by suppression (*F* < 1).

### Reaction times to rhymes (Suppression × Orthography × Distance)

Targets with matching orthography were detected faster than targets with nonmatching orthography [*F*(1, 34) = 33.45, *MSE* = 2,001,613, *p* < .0001, ${\eta}_p^2$ = .5]. We also observed a main effect of distance [*F*(2, 68) = 7.66, *MSE* = 173,090, *p* < .001, ${\eta}_p^2$ = .18], although the slowest responses were to targets at a distance of 1.

There was no overall effect of suppression (*F* < 1).

### Homophones (Suppression × Distance)

No significant effects emerged in the analysis of homophone detection RTs (suppression, *F* < 1; distance, *F* < 2).

### Recall

#### Rhymes

There was a significant effect of suppression on recall [*F*(1, 34) = 33.5, *MSE* = 115.0, *p* < .0001, ${\eta}_p^2$ = .5] and a significant effect of orthography [*F*(1, 34) = 22.9, *MSE* = 8.96, *p* < .0001, ${\eta}_p^2$ = .4], with trials containing orthographically dissimilar pairs leading to poorer recall. We also found a significant three-way interaction between suppression, orthography, and distance [*F*(2, 68) = 4.59, *MSE* = 1.41, *p* < .02, ${\eta}_p^2$ = .12], which is difficult to interpret.

#### Homophones

In the analysis of recall, the only significant effect was that of suppression [*F*(1, 34) = 8.85, *p* < .01, *MSE* = 63.7, *η*_p_^2^ = .21].

The recall data are shown in Table 1.

#### Combined

In the combined analysis of the recall data for all rhyme and homophone targets, only the effect of suppression was significant [*F*(1, 68) = 27.84, *MSE* = 64.6817, *p* < .0001]. The main effects of rhyme/homophone [*F*(1, 68) = 3.62, *MSE* = 8.4017, *p* = .061] and distance [*F*(2, 136) = 1.50, *MSE* = 0.239, *p* = .23], as well as the interaction between rhyme/homophone and suppression [*F*(1, 68) = 3.09, *MSE* = 7.1868, *p* = .083], all failed to reach significance.

## Discussion

In previous studies of rhyme and homophone judgments, the requirement to perform articulatory suppression has increased errors by a few percent at most. Often the effect has not been significant. However, here suppression had a huge effect on detection rates, especially for rhymes. Even the detection of adjacent rhymes dropped from 92% to 55% when the words were not orthographically similar and participants had to suppress. This decrease in accuracy for adjacent rhyme targets under suppression was mirrored by a large increase in false alarms to adjacent orthographic foils (up from 30% to 64%). In fact, with suppression, participants were more likely to judge that adjacent orthographically similar foils were rhymes than they were to detect orthographically different rhymes (64% vs. 54%). The high rate of errors to orthographic foils in rhyme detection suggests that participants must have been relying heavily on orthography, even in the absence of suppression. Note that although participants made more responses to orthographically similar foils than to orthographically dissimilar rhymes with suppression, orthographically dissimilar rhymes were still detected far above chance levels. If all phonological information had been lost, performance on orthographically dissimilar rhyme and homophone targets should have been the same as for orthographically dissimilar foils. That is, participants were still able to make limited use of phonological representations, but they were equally likely to base their responses purely on orthography.

This finding has parallels with the behavior of a short-term memory patient studied by Howard and Nickels (2005). They reported data from patient H.B., who had impaired auditory short-term memory but normal visual short-term memory. H.B. showed neither a phonological similarity effect nor a word-length effect. When tested on a rhyme judgment task, H.B. performed well by correctly classifying both orthographically similar and dissimilar rhymes (both 93%^1^) and correctly rejected 93% of orthographically dissimilar foils. However, H.B. only rejected 40% of orthographically similar foils. Likewise, when our participants performed articulatory suppression, they seemed to behave as though they had impaired phonological short-term memory and based their responses more on orthographic than on phonological information. The finding that articulatory suppression should mimic the effects of a phonological short-term memory deficit is exactly what would be expected on the basis of the working memory model.

Interestingly, we observed no effect of suppression on RTs for either rhyme or homophone detection. Superficially, this could be taken to imply that suppression has no effect on the overall difficulty of processing. However, given the large effect of suppression on errors, especially for rhyme detection, this null result is difficult to interpret. Perhaps it could be a consequence of a speed–accuracy trade-off, and the extra errors under suppression could have been to those trials that received the slowest responses without suppression. Alternatively, the main determinant of performance might be whether or not the item was available in memory, with suppression simply altering the probability that the item would be in memory rather than the quality or accessibility of the representation.

One further issue that warrants discussion is why suppression should have so little effect on homophone relative to rhyme judgments. Besner (1987) suggested that suppression impairs rhyme judgments because those judgments require phonological decomposition, which is not required for homophone judgments. Homophone judgments simply require a direct comparison of two identical phonological representations. But is there any relationship between phonological decomposition and serial recall? Superficially it seems unlikely that suppression could interfere with serial recall by virtue of an effect on phonological decomposition, since there is no necessary requirement for decomposition in the serial-recall task. However, the pattern of errors in serial recall of phonologically confusable lists indicates that the information is being stored in terms of units such as onsets and rimes. Phonological confusions in immediate serial recall primarily involve exchanges in which the onsets swap with other onsets and rimes with rimes (Fallon, Groves, & Tehan, 1999; Nimmo & Roodenrys, 2004). Although the immediate serial-recall task itself may not require decomposition of phonological representations into onsets and rimes, the evidence suggests that this kind of structured representation is exactly what underpins the operation of the phonological store.

The present data help reconcile the apparently contradictory results from serial-recall and phonological judgment tasks. Even when performing immediate serial recall under articulatory suppression, participants can still perform rhyme and homophone judgments at better than chance levels. Thus, suppression does not block phonological recoding completely. However, suppression does have a dramatic effect on performance, even for homophones. In fact, phonological representations appear to be degraded to such an extent that performance is reduced to levels that might be expected of patients with a phonological short-term memory deficit.

## Appendix

**Table 2:** Stimuli for rhyme detection

	Rhymes					Foils		
	–Orthography		+Orthography			+Orthography		
	TOUGH	STUFF	SCENT	RENT	BRUISE	GUISE	GROW	COW
	FRIGHT	BITE	LIFT	GIFT	LEAF	DEAF	HOOT	SOOT
	LORD	BORED	LAMP	DAMP	COUP	SOUP	WEIGHT	HEIGHT
	DRAIN	CANE	BAND	SAND	GIVE	FIVE	BREAST	FEAST
	WAIT	DATE	LUMP	THUMP	THROUGH	ROUGH	GRASS	MASS
	ROPE	SOAP	FOND	POND	THEY	KEY	BONE	DONE
	HIGH	WHY	LEND	BEND	YOUTH	MOUTH	STEAK	LEAK
	CHORD	SOARED	HINGE	BINGE	FOUR	HOUR	SEW	PEW
	FENCE	DENSE	MINT	TINT	GHOST	FROST	PLOUGH	DOUGH
	THEME	BEAM	LAND	HAND	LOVE	DROVE	LOSE	CLOSE
	YACHT	CLOT	WEALTH	HEALTH	WHEAT	SWEAT	SIEVE	GRIEVE
	HEAT	SHEET	PALM	CALM	GREAT	SEAT	COUGH	BOUGH
	RAFT	DRAUGHT	CAMP	RAMP	GONE	PHONE	WOOD	MOOD
	SHOE	GLUE	TANK	SANK	FOOT	ROOT	FOOD	HOOD
	BLUE	FLEW	PINK	LINK	LEAR	BEAR	COMB	WOMB
Frequency	17,794		8,788			48,434		
ELP RT	612		590			625		

Frequencies are from the SUBTLEX-UK corpus (Van Heuven et al., 2014). ELP RT is the mean pronunciation latency in milliseconds from the English Lexicon Project (Balota et al., 2007).

**Table 3:** Stimuli for homophone detection

	Homophones				Foils			
	BEACH	BEECH	PREY	PRAY	ARE	AIR	BORE	BOW
	MALE	MAIL	BLUE	BLEW	MEAN	MOON	BOAST	BOOST
	POUR	PORE	CAUGHT	COURT	DARE	DEAR	HOME	HUM
	ROUTE	ROOT	TEA	TEE	SNEAK	SNAKE	FLEW	FLEA
	SEA	SEE	SAIL	SALE	FERRY	FURY	EAR	OAR
	PAIL	PALE	WRITE	RIGHT	LICK	LIKE	LIAR	LAIR
	WEIGH	WAY	THEIR	THERE	MILK	MILL	NEW	NO
	SORE	SAW	THAI	TIE	FEAR	FARE	LOAD	LEAD
	STAIR	STARE	STEEL	STEAL	WEAR	WERE	CROWD	CRUDE
	BREAK	BRAKE	TOW	TOE	BURN	BARN	CHAIR	CHOIR
	EARN	URN	HEAR	HERE	PEACH	POACH	FIRE	FEAR
	MIGHT	MITE	KNOWS	NOSE	LAMP	LUMP	TAME	TEAM
	SOME	SUM	PEAR	PAIR	RAID	RIDE	NUT	NOT
	MAID	MADE	SOLE	SOUL	PEA	PIE	TOUCH	TORCH
	CELL	SELL	SEW	SO	WEAK	WAKE	BEAR	BEER
Frequency	88,151				84,254			
ELP RT	613				600			

Frequencies are from the SUBTLEX-UK corpus (Van Heuven et al., 2014). ELP RT is the mean pronunciation latency in milliseconds from the English Lexicon Project (Balota et al., 2007).

## Footnotes

1 93% corresponds to only a single error.
